# Pharmacokinetics, Tissue Distribution, and Elimination of Three Active Alkaloids in Rats after Oral Administration of the Effective Fraction of Alkaloids from *Ramulus Mori*, an Innovative Hypoglycemic Agent

**DOI:** 10.3390/molecules22101616

**Published:** 2017-09-26

**Authors:** Shuang Yang, Jiaqi Mi, Zhihao Liu, Baolian Wang, Xuejun Xia, Renyun Wang, Yuling Liu, Yan Li

**Affiliations:** 1 State Key Laboratory of Bioactive Substance and Function of Natural Medicines, Beijing Key Laboratory of Non-Clinical Drug Metabolism and PK/PD Study, Department of Drug Metabolism, Institute of Materia Medica, Chinese Academy of Medical Sciences & Peking Union Medical College, Beijing 100050, China; sylvia.mi@hotmail.com (J.M.); liuzhihao12399@126.com (Z.L.); yanli@imm.ac.cn (Y.L.); 2 Department of Pharmaceutical Analysis, School of Medicine and Pharmacy, Ocean University of China, Qingdao 266003, China; yangshuang@ouc.edu.cn; 3 Department of Pharmaceutics, Institute of Materia Medica, Chinese Academy of Medical Sciences & Peking Union Medical College, Beijing 100050, China; xjxia@imm.ac.cn (X.X.); wry@imm.ac.cn (R.W.); ylliu@imm.ac.cn (Y.L.)

**Keywords:** *Ramulus Mori*, alkaloids, pharmacokinetics, tissue distribution, excretion, biotransformation

## Abstract

In this study, we systematically investigated the plasma pharmacokinetics, tissue distribution, and elimination of three active alkaloids after oral administration of the effective fraction of alkaloids from *Ramulus Mori* (SZ–A)—an innovative hypoglycemic agent—in rats. Moreover, the influences of other components in SZ–A on dynamic process of alkaloids were explored for the first time. The results showed that 1-deoxynojirimycin (DNJ), fagomine (FGM) and 1,4-dideoxy-1,4-imino-d-arabinitol (DAB) exhibited nonlinear pharmacokinetics following oral administration of SZ–A (40–1000 mg/kg). The prolonged t_1/2_ and greater area under concentration-time curve (AUC) versus time (AUC_0–t_) of DNJ for SZ–A than for purified DNJ has been observed after both oral and intravenous administration. It was found that other components in SZ–A could enhance the absorption of DNJ through the intestinal barrier. The major distribution tissues of DNJ, FGM, and DAB were the gastrointestinal tract, liver, and kidney. Three alkaloids were mainly excreted into urine and feces, but less into bile. Interestingly, the excess excretion of FGM was revealed to be partly due to the biotransformation of other components in SZ–A via gut microbiota. These information provide a rational basis for the use of SZ–A in clinical practice.

## 1. Introduction

Diabetes mellitus (DM) is a serious disease that can cause a lot of life-threatening health complications [1]. International Diabetes Federation (IDF) estimated that there would be 642 million persons with DM in 2040 [2]. Postprandial blood glucose control is found to be crucial in the therapy of diabetes. α-glycosidase inhibitors, such as acarbose and miglitol, are widely used for decreasing postprandial hyperglycemia [3]. However, their gastrointestinal adverse effects were observed during monotherapy or drug combination [4,5].

The effective fraction of alkaloids from traditional Chinese herbal medicine *Ramulus Mori* (Chinese name: Sangzhi), abbreviated to SZ–A, was developed to be an innovative hypoglycemic agent. Its phase III clinical trials for the treatment of type 2 DM have been performed in China (registered at https://db.yaozh.com/linchuangshiyan, number: CTR20140034, CTR20140569). SZ–A possessed a potent inhibitory activity to α-glucosidase and similar mechanism with acarbose [6,7]. It could decrease the glycated hemoglobin concentration (HbA1c), 1 h and 2 h postprandial plasma glucose levels, but not the fasting plasma glucose levels. Furthermore, the side effects of SZ–A, such as flatulence and diarrhea, was obviously less than acarbose because of its lower inhibition to amylase [8,9].

**Figure 1:**
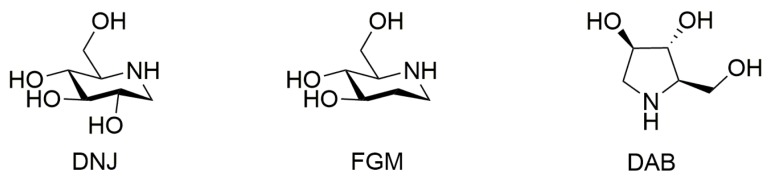
Chemical structures of DNJ, FGM, and DAB.

The polyhydroxy alkaloids (50% or more by weight), represented by 1-deoxynojirimycin (DNJ), fagomine (FGM) and 1,4-dideoxy-1,4-imino-d-arabinitol (DAB) (Figure 1), were known to be the major hypoglycemic active constituents in SZ–A [8]. It was reported that only 1% of the ingested DNJ (110 mg/kg) could be incorporated into the rat plasma [10], and DNJ was considered to act on the small intestine by blocking the digestion of complex carbohydrates. In contrast, it was found that DNJ could be absorbed and eliminated rapidly after oral administration of SZ–A in our previous study [11]. To our knowledge, the oral bioavailability of DAB (2.3 mg/kg) was 89% [12]. However, we still lacked some basic knowledge of the active alkaloids in SZ–A, including pharmacokinetics, tissue distribution, and excretion in vivo in experimental animals. Additionally, it was reported that the pharmacokinetics of DNJ could be impacted by soluble dietary fiber and Radix Pueraria flavonoids [13,14]. As we know, there were other components accounting for about 50% of the total SZ–A extract. Thus, the influences of other components in SZ–A on the dynamic process of the three alkaloids needs to be clarified.

The purpose of this study is to determine the in vivo pharmacokinetic profiles and characteristics of DNJ, FGM, and DAB after oral administration of SZ–A. In this study, the pharmacokinetics, tissue distribution, and excretion of three representative alkaloids were investigated with the liquid chromatography–tandem mass spectrometry (LC-MS/MS) method. The effects of other components on the oral absorption of DNJ and excretion of FGM was also confirmed by in situ single-pass intestinal perfusion and in vitro incubation with rat intestinal homogenate and with caecal microbiota cultures. This was the first time a systematic study on the pharmacokinetics, distribution, and elimination of DNJ, FGM, and DAB from SZ–A has been conducted. We hope the results would be useful for the physiologically-based pharmacokinetic modeling to simulate the performance of SZ–A in humans and provide reliable experimental data for its safe and effective use in clinical practice.

## 2. Results

### 2.1. Pharmacokinetic Studies of Three Alkaloids

**Figure 2:**
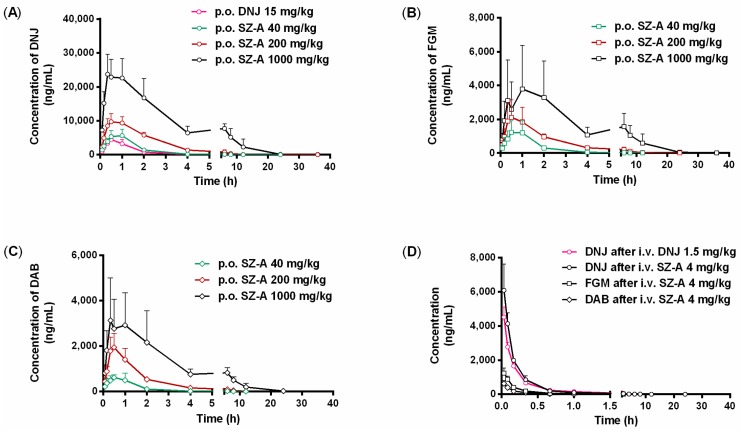
Mean plasma concentration-time curves s in rats after oral ((**A**) DNJ; (**B**) FGM; (**C**) DAB) and intravenous (**D**) administration of SZ–A and purified DNJ (*n* = 5).

Plasma concentration-time curves of DNJ, FGM, and DAB in rats after oral (40, 200, 1000 mg/kg) and intravenous (4 mg/kg) administrations of SZ–A are depicted in Figure 2 and the major pharmacokinetic parameters are summarized in Table 1. The concentration-time curves of three alkaloids after oral administration of SZ–A at high dose showed the phenomenon of a double-peak in the plasma profile, which was not shown at middle and low doses. DNJ, FGM, and DAB reached their maximum plasma concentrations (C_max_) within 0.67, 1.30, and 0.67 h after oral administration of SZ–A, demonstrating the rapid absorption of alkaloids from the gastrointestinal tract. Half-life (t_1/2_) and mean residence time (MRT) values of all three active analytes were prolonged with dose escalation. Additionally, the five-fold increase in dosage led to only 2.4- to 4.3-fold increase in the area under concentration-time curve (AUC) versus time (AUC_0–t_) of the major constituents. The oral absolute bioavailability (F) was found to be 72.41%, 77.50%, and 78.23% for DNJ, FGM, and DAB when the dose of SZ–A was 40 mg/kg in rats. Moreover, the absolute bioavailability values of the three alkaloids remarkably decreased with increasing dose. These findings revealed nonlinear pharmacokinetics of the three alkaloids at the investigated dosage of SZ–A in rats.

Also, Figure 2 showed the plasma concentration-time curves of DNJ in rats after oral administration of purified DNJ at 15 mg/kg, as well as 1.5 mg/kg intravenously. The pharmacokinetic parameters listed in Table 1 showed that C_max_ and time to maximum concentration (T_max_) values of DNJ were similar between the DNJ group and SZ–A Group after oral administration at equivalent dose of DNJ. However, compared with the DNJ group, t_1/2_ of DNJ in the SZ–A group was slightly delayed from 0.88 h to 1.30 h (*p* < 0.05). Meanwhile, the average values of AUC in the SZ–A group were found to be 1.5-fold (oral administration) and 1.2-fold (intravenous injection) higher than that in the DNJ group. Additionally, the total body clearance (CLz) value of DNJ was smaller in the SZ–A group. These results suggested that the components in SZ–A could impact the exposure of DNJ in rats.

### 2.2. Impacts of Other Components in SZ–A on Absorption of DNJ

**Figure 3:**
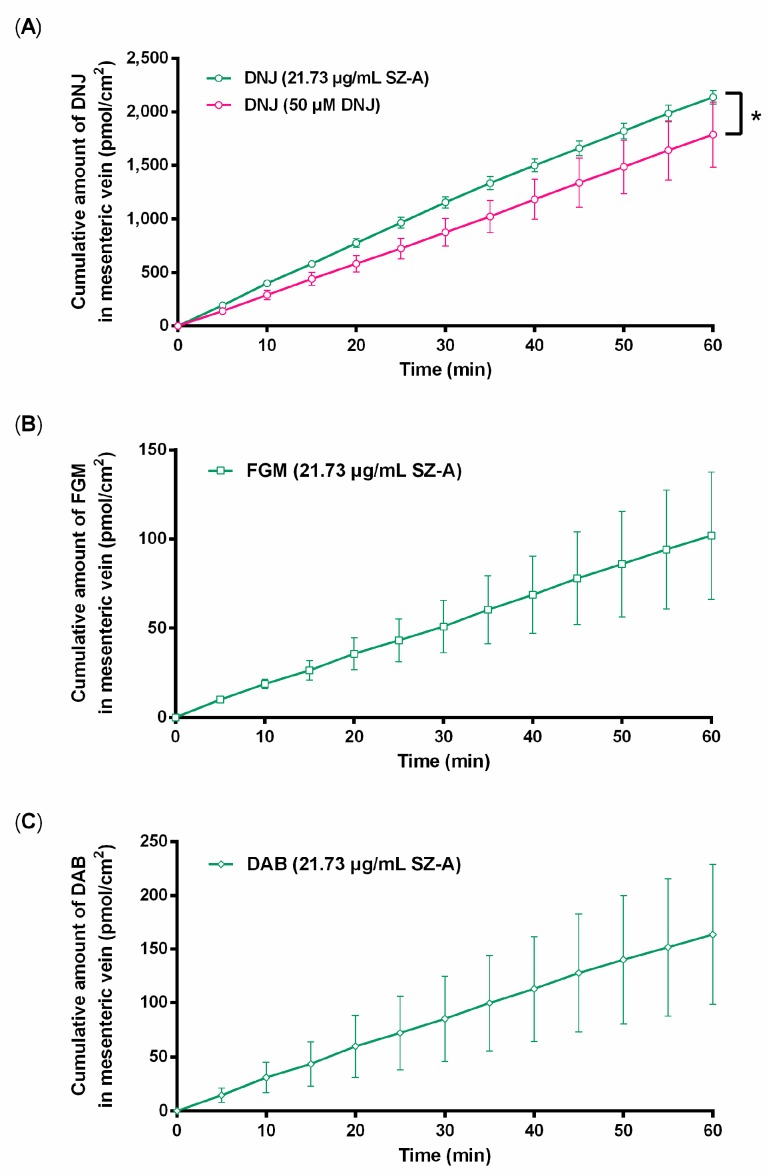
Cumulative amount of DNJ (**A**); FGM (**B**); and DAB (**C**) in the mesenteric vein after rat jejunum perfusion (*n* = 5). The statistically significant difference is between SZ–A at 21.73 μg/mL and DNJ (50 μM), * *p* < 0.05.

To determine the reason for the higher exposure of DNJ in rats after administration of SZ–A, the intestinal absorption of the components was investigated through in situ single-pass intestinal perfusion of SZ–A (21.73 μg/mL, containing 50 μM DNJ, 9.5 μM FGM, 7.8 μM DAB) and purified DNJ (50 μM). Here, the P*_lumen_* of propranolol and atenolol agreed with previous values obtained in single-pass intestinal perfusion in rats [15], which suggested that the assay was reliable. The cumulative amount of three alkaloids in mesenteric plasma was increased time–dependently (Figure 3), suggesting that three compounds could be absorbed into blood through the intestinal barrier. However, the cumulative amount per unit of area of DNJ absorbed into the mesenteric venous blood from SZ–A (2136.1 pmol/cm^2^) was higher than that in the DNJ group (1787.1 pmol/cm^2^) by 60 min. These results demonstrated that the absorption of DNJ was enhanced by the other components in SZ–A, which was responsible for the higher exposure of DNJ in the SZ–A group in the pharmacokinetic study.

### 2.3. Tissue Distribution

**Figure 4:**
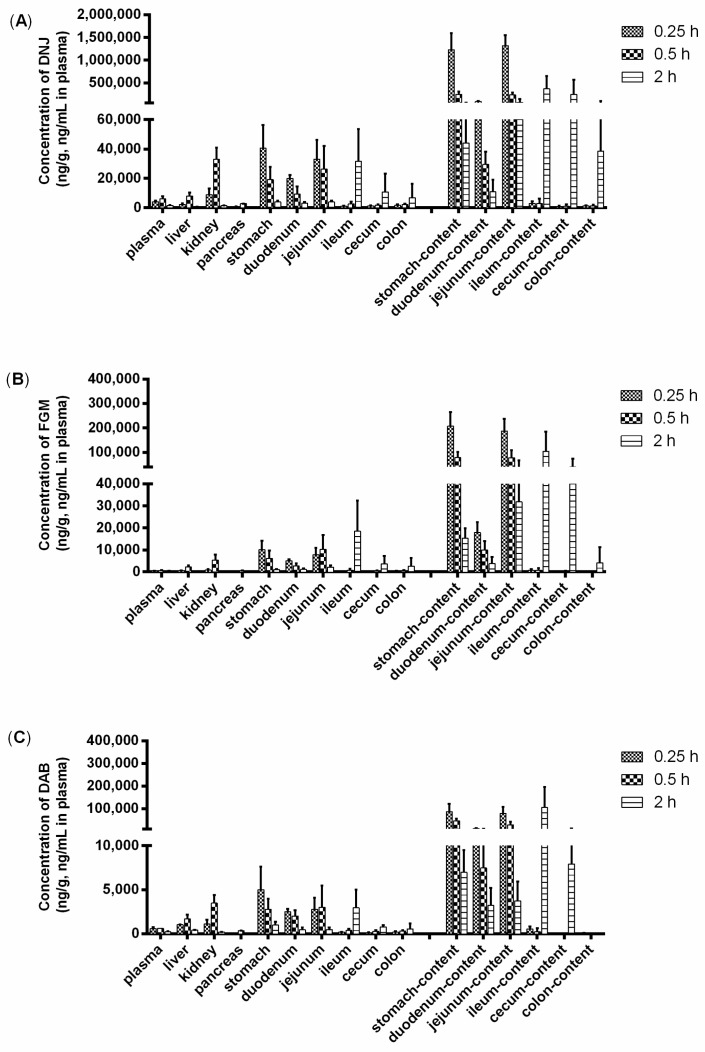
Tissue concentration of DNJ (**A**), FGM (**B**); and DAB (**C**) in rats after oral administration of SZ–A at 40 mg/kg (*n* = 3).

The concentrations of three alkaloids in rat tissues collected at 0.25, 0.5, and 2 h following a single oral administration of SZ–A (40 mg/kg) were depicted in Figure 4. The results of the present study indicated that DNJ, FGM, and DAB underwent a rapid distributed into tissues within the time course examined, and no long-term accumulation of the compounds in tissues was observed.

The major distribution tissues of DNJ, FGM, and DAB were similar. The maximum tissue concentration of all of the analytes was observed in gastrointestinal tract. The concentration of three alkaloids in gastrointestinal tract was found to be highest at 0.25 h post-dose in the stomach and duodenum, but at 2 h in the ileum, cecum, and colon. Additionally, the jejunum had similar concentration of three analytes at 0.25 h and 0.5 h. By 2 h post-dose, the tissue to plasma ratio (T/P) values of three analytes was almost decreased with time in the stomach, duodenum, and jejunum, and increased in the ileum, cecum, and colon, simultaneously. These results, which were consistent with the concentration changes of three alkaloids in the gastrointestinal content, may be mainly attributed to the oral mode of administration. In addition, all three analytes were mainly distributed in abundant blood-supply tissues, such as the kidney and liver, besides the gastrointestinal tract. The highest tissue concentration of DNJ, FGM, and DAB in the kidney, liver, and pancreas was detected within 0.5 h of SZ–A administration. Meanwhile, T/P value of DNJ, FGM, and DAB in the pancreas was relatively lower over the range of 0.06–0.47, 0.18–1.13, and 0.09–0.59. The results implied that the distribution of the compounds depended on the blood flow or perfusion rate of the organ.

### 2.4. Excretion of Three Alkaloids

**Figure 5:**
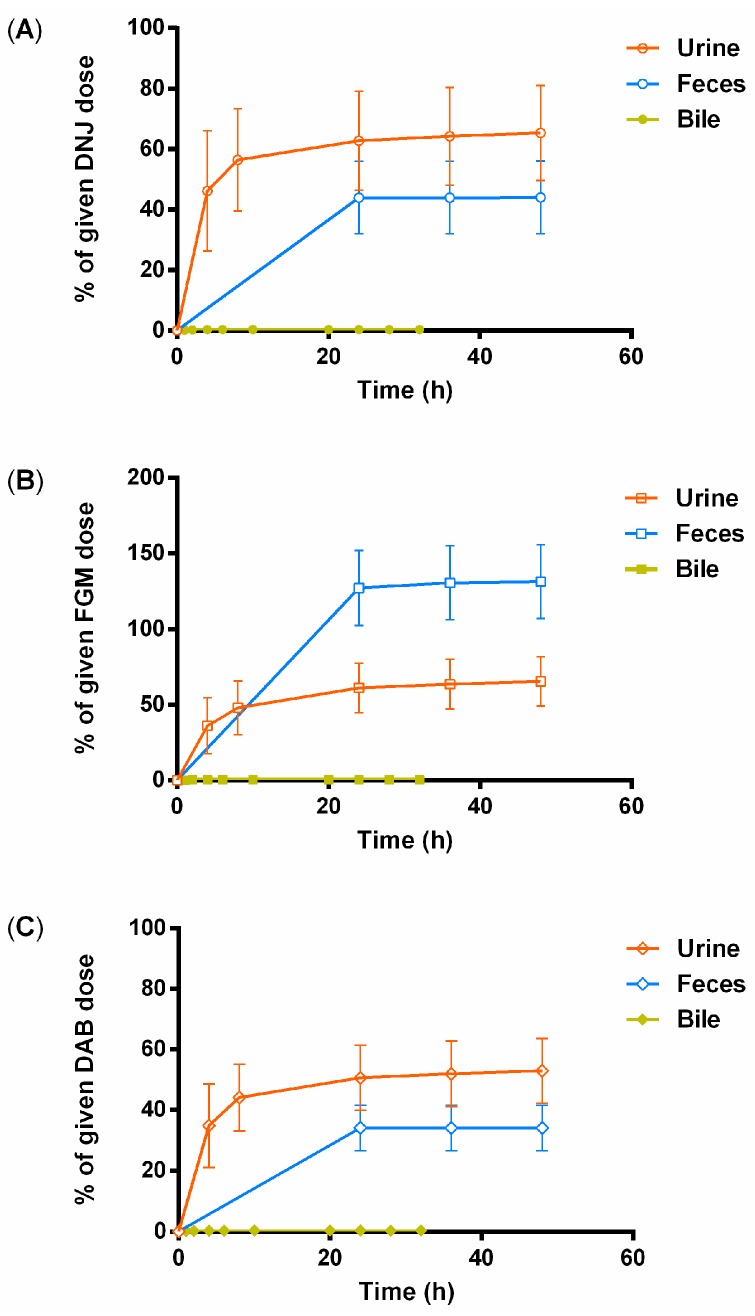
Cumulative excretion percentage of DNJ (**A**), FGM (**B**), and DAB (**C**) in urine, feces, and bile after an oral administration of SZ–A at 40 mg/kg in rats (*n* = 5).

The cumulative amount of the three active alkaloids excreted into urine and feces after oral administration of SZ–A at 40 mg/kg are illustrated in Figure 5. Over a 24 h period, 65.32% of DNJ was examined in urine and 43.97% in feces. The urinary and fecal excretion of FGM was 65.36% and 131.4%, respectively. Additionally, 52.91% of DAB was excreted into urine and 34.05% into feces. Furthermore, the findings suggested that biliary excretion was not the major route of DNJ, FGM, and DAB, indicated by the recoveries of 0.29%, 0.50%, and 0.35% in bile within 32 h. Interestingly, the total excretive amount of FGM was found to be about two-fold of the oral dose. It was inferred that the excess excretion of FGM into feces was biotransformed by the other components in SZ–A.

### 2.5. Biotransformation of Other Components in SZ–A

**Figure 6:**
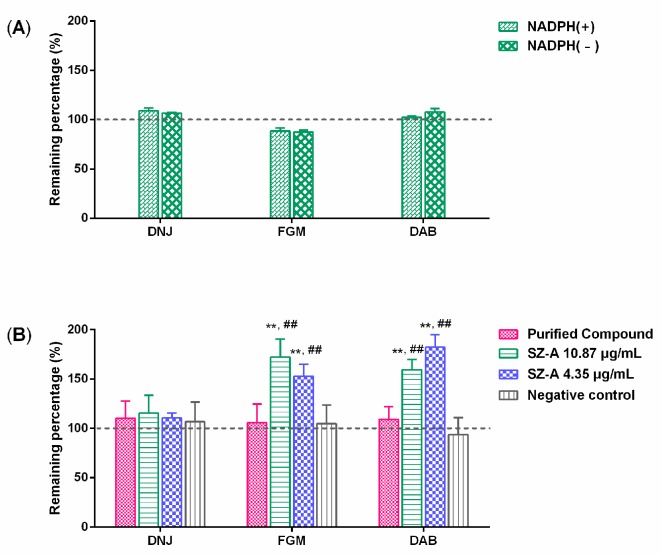
Remaining percentage of DNJ, FGM, and DAB from SZ–A in rat intestinal homogenate (**A**) and rat caecal microbiota cultures (**B**) (*n* = 3). Compared with the purified DNJ group, ** *p* < 0.01; compared with the negative control group, ^##^ *p* < 0.01.

To determine whether intestinal metabolizing enzymes or the gut microbiota was responsible for the higher exposure of DNJ and the excess excretion of FGM in feces, SZ–A was incubated with intestine homogenate and rat caecal microbiota cultures and then the three alkaloids were quantified. As shown in Figure 6A, the amount of DNJ, FGM, and DAB in SZ–A (10.87 μg/mL) was not changed significantly in the incubation of rat intestinal homogenate with/without NADPH generation. However, DNJ, FGM, and DAB were determined to be 115.5%, 172.0%, and 159.3% of initial amount after incubation with 10.87 μg/mL SZ–A (containing 25 μM DNJ, 4.7 μM FGM, 3.9 μM DAB) and rat gut microbiota cultures for 2 h, as well as 110.6%, 152.8%, and 182.2% with 4.35 μg/mL SZ–A (containing 10 μM DNJ, 1.9 μM FGM, 1.6 μM DAB) (Figure 6B). Meanwhile, the amount of three alkaloids in the incubation samples with boiled rat caecal microbiota cultures was not increased. Therefore, the metabolism of SZ–A by the gut microbiota resulted in the excess excretion of FGM. Additionally, the slight increase of DNJ in the gut microbiota cultures indicated that the biotransformation of other components in SZ–A might be another reason for the higher exposure of DNJ in rats after dosing SZ–A than purified DNJ.

## 3. Discussion

As we know, α-glucosidase inhibitor could prevent digestion of carbohydrates to absorbable monosaccharides by inhibiting α-glucosidase on the small intestinal brush border to decrease the blood glucose levels. Therefore, the small intestine was considered to be the main acting site of SZ–A. However, it was not confirmed whether there was also another active mechanism of SZ–A in vivo since DNJ, FGM, and DAB were reported to be absorbable in rats and other components (about 50%) in SZ–A existed, except the three major active alkaloids [11]. It is well known that pharmacokinetic studies are helpful to define and understand a variety of events related to pharmacologic efficacy. The aim of the current study was to characterize the fate of DNJ, FGM, and DAB from SZ–A in rats, and also to explore the influence of other components from SZ–A on the exposure of three alkaloids.

As major active ingredients—DNJ, FGM, and DAB—were found to be well absorbed following oral administration with good absolute bioavailability, and then rapidly distributed to the liver and kidneys besides the gastrointestinal tract. It is reported that DNJ—the potent inhibitor of acid α-glucosidase and α-1,6-glucosidase—could decrease the rate of glycogenolysis caused by glucagon in rats [16,17,18]. FGM—the inhibitor of isomaltase, α-, β-glucosidase, and α-, β-galactosidase—could lower blood glucose in a dose-dependent manner without stimulating insulin secretion in rats [19,20,21]. DAB displayed an anti-hyperglycemic effect by inhibiting glycogen phosphorylase in mice [22]. The relatively high distribution of three active alkaloids in liver suggested that the inhibition of glycogenolysis might be an alternative hypoglycemic mechanism. Hence, it was inferred that SZ–A played the hypoglycemic role not only by decreasing the absorption of glucose through inhibiting the intestinal α-glucosidase enzymes, but also by decreasing the glycogenolysis in vivo through inhibiting the enzymes which were responsible for the hepatic glucose production. Moreover, the relatively high distribution in kidney demonstrated that the kidney might be the primary excretion organ of DNJ, FGM, and DAB. This was confirmed by the urinary excretion of three alkaloids (more than 50% of dosage).

In the present study, the double-peak of mean plasma concentration-time curves of the three alkaloids was observed in the high-dose group (Figure 2). Since less than a 0.5% cumulative amount of three active alkaloids of the three alkaloids was determined in bile, the phenomenon of double-peak behavior could not be due to enterohepatic recycling. These results implied that the absorption could be saturated over 1000 mg/kg SZ–A. Meanwhile, the MRT value of each tested alkaloid was prolonged significantly, which could be associated with the ability of elimination saturation.

In the current study, in situ single-pass intestinal perfusion, as a robust tool for simulating real in vivo conditions following oral drug administration [23], was employed to evaluate the influence of other components in SZ–A on the exposure of DNJ. It was found that the absorption rate of DNJ was higher when SZ–A was applied for in situ intestinal perfusion. Additionally, in pharmacokinetic studies, the clearance rate of DNJ was found to be significantly slowed after intravenous injection of SZ–A. Thus, it was deduced that the higher exposure of DNJ in rats after oral administration of SZ–A could be partly due to the higher absorption rate and lower clearance rate. However, further study on the absorption and elimination mechanism of DNJ is needed to determine which constituent, and how it affects the exposure of DNJ from SZ–A.

Interestingly, it was found that the total excretion of FGM from urine and feces was about two-fold of the given dose. Beyond urinary excretion of the bio-accessible amount, fecal excretion reached up to 131.41% of oral administration dosage. It is reported that the gut microbiota, considered as a “hidden organ”, and intestinal enzymes played very important role in drug metabolism in some cases [24,25,26]. In fact, there were stereoisomers and glycosides of three active alkaloids in SZ–A**,** such as 3-epi-fagomine, 2-*O*-(α-d-galactopyranosyl)-1-deoxynojirimycin, 6-*O*-(β-d-glucopyranosyl)-1-deoxynojirimycin and 1,4-dideoxy-1,4-imino-(2-*O*-β-d-glucopyranosyl)-d-arabinitol [8], which might be biotransformed to be DNJ, FGM, and DAB. The results illustrated that the unabsorbed SZ–A was maintained in the alimentary tract for a while and might be biotransformed to DNJ, FGM, and DAB by gut microbiota, but not by intestinal enzymes. However, the caecal microbiota cultures incubation assay in vitro was so limited for studying the metabolic mechanism. Hence, human gastrointestinal microbiota and more in vivo models, such as germ-free, pseudo-germ-free, and gnotobiotic animals should be used in further research [27].

## 4. Materials and Methods

### 4.1. Chemical and Reagents

DNJ (purity >98.0%) and SZ–A (lot number: 20130717-2), containing 37.5% of DNJ, 6.4% of FGM, and 4.8% of DAB, was kindly provided by the Department of Pharmaceutics (Institute of Materia Medica, Chinese Academy of Medical Sciences, Beijing, China). Miglitol (internal standard, IS) was purchased from J and K Scientific Ltd., (Beijing, China). FGM (purity >98.0%) was obtained from Medchem Express Co., Ltd., (Princeton, NJ, USA). DAB∙HCl (purity >98.0%), propranolol, atenolol, β-nicotinamide adenine dinucleotide phosphate (β-NADP) hydrate, d-glucose 6-phosphate (G-6-P) disodium salt hydrate, glucose-6-phosphate dehydrogenase (G-6-PDH), and ammonium hydroxide solution were products of Sigma-Aldrich Co., Ltd., (St. Louis, MO, USA). Tris(hydroxymethyl)aminomethane (Tris) was obtained from Sinopharm Chemical Reagent Co., Ltd., (Beijing, China). Anaerobic medium was purchased from Beijing Land Bridge Technology Co., Ltd., (Beijing, China). Acetonitrile and methanol were of HPLC grade (Merck KGaA, Darmstadt, Germany). Ultrapure water was prepared by a Milli-Q Reagent water system (Millipore, Billerica, MA, USA). All other chemicals were of analytical reagent grade and commercially available.

### 4.2. Animals

All animal protocols were approved by Institutional Animal Care and Welfare Committee of the Chinese Academy of Medical Sciences (Beijing, China; permission number: SYXK 2009-0004, SYXK 2014-0023) and the Tab of Animal Experimental Ethical Inspection number was 00001228. Male Sprague-Dawley (SD) rats (180–220 g) were obtained from Beijing Vital River Experimental Animal Co., Ltd. (Beijing, China, approval number: SCXK 2012-0001), and acclimated for seven days on a 12 h light/12 h dark cycle at 22 ± 2 °C, 60% relative humidity. The animals were allowed free access to water and chow diet. Rats were fasted for 12 h with water ad libitum before oral dosing or intestinal perfusion.

### 4.3. Pharmacokinetic Study

The oral dosing solutions used for all animal studies were prepared by dissolving in water. Thirty rats weighed 250–270 g after acclimation were randomly divided into six groups of five animals each. For oral administration, rats were treated with SZ–A (40, 200, 1000 mg/kg) or purified DNJ (15 mg/kg) dissolve in water. Blood samples (about 0.25 mL) were collected via the postorbital venous plexus veins under ether anesthesia into heparinized polythene tubes before dosing and at 0.08, 0.17, 0.33, 0.50, 1, 2, 3, 4, 6, 8, 12, 24 and 36 h after drug administration. For intravenous dosing, rats were given SZ–A (4 mg/kg) or DNJ (1.5 mg/kg) dissolved in normal saline solution through the tail vein. Then blood samples were collected at 0.03, 0.08, 0.17, 0.33, 0.67, 1, 2, 4, 6, 8, 12, 24, and 36 h under similar conditions. Considering the physical limit of blood in rats, during the process of the pharmacokinetic experiment, each rat was received 0.25 mL of sterile isotonic saline at each time point after blood sampling for compensation of body fluid loss, and the blood loss was also corrected at a definite degree. Plasma was separated by centrifuging the blood samples at 1660× *g* for 5 min and stored at −20 °C until LC-MS/MS analysis. Data fitting and pharmacokinetic parameter estimates were carried out using non–compartmental analysis via WinNonlin Software (version 6.1, Pharsight Corporation, Mountain View, CA, USA). F was calculated according to the formula: F (%) = (AUC oral/AUC i.v.) × (dose i.v./dose oral) × 100.

### 4.4. Single-Pass Intestinal Perfusion In Situ

The surgical procedures were similar to those described elsewhere [28]. The Hank’s balanced salt solution (HBSS, pH = 7.4) with SZ–A (21.73 μg/mL) or DNJ (50 μM) at 37 °C was infused into the jejunum at 0.3 mL/min individually. Phenol Red (50 μM) was added to serve as a nonabsorbable marker for measuring water flux. The blood from mesenteric vein and perfusate were collected at five-minute intervals. The plasma was separated by centrifugation and perfusate were immediately frozen at −20 °C for LC-MS/MS analysis. Atenolol and propranolol, the low- and high-permeability markers, were used to check the integrity of the intestinal membrane.

### 4.5. Tissue Distribution

Three groups of rats (three rats in each group) were administered with a single oral dose of SZ–A at 40 mg/kg. Rats each were sacrificed and various tissues, including liver, kidney, pancreas, stomach, duodenum, jejunum, ileum, cecum, colon, and the content in the gastrointestinal tract were collected at 0.25, 0.5, and 2 h time points. One additional rat was sacrificed predose to provide control tissues for analysis. After removing blood or content and weighing, each sample was individually homogenized with normal saline solution (1:3, *w*/*v* for tissue; 1:9, *w*/*v* for content) using a tissue homogenizer (Polytron PT1600E, Kinematica, Luzern, Switzerland) and stored at −20 °C until LC-MS/MS analysis.

### 4.6. Excretion

Five rats were kept in the metabolic cages with free access to water and food 2 h post dose. Urine was collected before dosing and at 0–4, 4–8, 8–24, 24–36, and 36–48 h, and feces was simultaneously collected before dosing and at 0–24, 24–36, and 36–48 h after oral dosing of SZ–A (40 mg/kg). Feces samples were dried and grinded into powder, then homogenized with normal saline solution (1:9, *w*/*v*). Additionally, the bile duct of rats under ether anesthesia were cannulated with a PE-10 tube (Portex Ltd., Kent, UK). Bile was collected before dosing and at 0–1, 1–2, 2–4, 4–6, 6–10, 10–20, 20–24, 24–28, and 28–32 h following oral administration of SZ–A (40 mg/kg). All urine, feces, and bile samples were stored at −20 °C until LC-MS/MS analysis.

### 4.7. In Vitro Incubation of SZ–A with Rat Intestinal Homogenate and Caecal Microbiota Cultures

Pooled rat intestinal homogenate from six males was prepared using a tissue homogenizer with ice cold normal saline solution at a proportion of 1:3 (*w*/*v*). SZ–A was incubated with rat intestinal homogenate (5 mg protein/mL) with/without a NADPH-generating system (0.011 M β-NADP, 0.1 M glucose 6-phosphate, 10 U/mL glucose-6-phosphate dehydrogenase) in a 37 °C water bath for 120 min. Meanwhile, caecal content was collected and mixed with anaerobic medium (1:30, *w*/*v*) to prepare the cultures under nitrogen atmosphere at 37 °C for 120 min. SZ–A and rat caecal microbiota cultures were incubated at a total volume of 200 μL with the boiled samples as the negative control, concomitantly. The incubation was stopped at 2 h by adding two volumes of methanol–acetonitrile (1:3, *v*/*v*). Triplicate supernatants were taken by centrifugation at 18,880× *g* for 5 min and stored at −20 °C until LC-MS/MS analysis. The results were expressed as the percentage of the concentration at 0 min.

### 4.8. Sample Preparation and LC-MS/MS Analysis

The plasma, feces, urine, and bile samples (100 μL) were added to 1 μL of Tris aqueous solution (2 M), 10 μL of IS working solution (2 μg/mL), and 190 μL methanol–acetonitrile (25:75, *v*/*v*). The incubation supernatant samples (300 μL) were mixed with 1 μL Tris aqueous solution (2 M) and 10 μL of IS working solution (2 μg/mL). All mixtures were vortexed for 30 s followed by centrifugation at 18,880× *g* for 5 min, twice.

Then the samples were analyzed using the reported LC-MS/MS method as described in our previous study, and no interference was observed at the retention time of the analytes and IS in samples collected before dosing [11]. Briefly, the LC-MS/MS instrument consisted of a 1260 HPLC system (Agilent, Santa Clara, CA, USA) and API4000 triple quadrupole mass spectrometer (Applied Biosystems Sciex, Toronto, ON, Canada) with an electrospray ionization (ESI) source and Analyst software (version 1.5.2, Applied Biosystems Sciex, Toronto, ON, Canada) for data acquisition and processing. DNJ, FGM, DAB, and IS were separated in a Xbridge amide™ column (3.5 μm, 4.6 mm × 150 mm; Waters, Milford, MA, USA) with a gradient mobile phase of acetonitrile-water (0.1% ammonium hydroxide) at a flow rate of 0.7 mL/min. An ESI source was used in the positive ion mode. The optimized mass spectrometric condition was as follows: ion spray voltage, 5500 V; collision gas, 6 psi; curtain gas, 20 psi; ion source gas 1 (GS1), 45 psi; ion source gas 2 (GS2), 45 psi; temperature, 450 °C. The multiple reaction monitoring (MRM) transitions of DNJ, FGM, DAB, and IS were *m*/*z* 164.0→110.1 (declustering potential (DP) 50 V, collision energy (CE) 22 V, collision cell exit potential (CXP) 10 V), 148.0→112.1 (DP 66 V, CE 21 V, CXP 10 V), 134.0→98.0 (DP 60 V, CE 20 V, CXP 10 V) and 208.0→146.1 (DP 50 V, CE 27 V, CXP 10 V), respectively.

### 4.9. Data Analysis

All data were expressed as mean with standard deviation (mean ± SD). The statistical analyses were conducted using one-way ANOVA and *p* values less than 0.05 were considered statistically significant.

## 5. Conclusions

In general, DNJ, FGM, and DAB, which were rapidly absorbed and distributed to the gastrointestinal tract, liver, and kidney, and then eliminated by renal and fecal excretion, exhibited nonlinear pharmacokinetics after oral administration of SZ–A (40–1000 mg/kg). Furthermore, other components in SZ–A could affect the absorption and elimination process of alkaloids. These results may be useful for the clinical practice of SZ–A, which can also provide reliable scientific data for ameliorating drug treatment regimens.

## Figures and Tables

**Table 1:** Pharmacokinetic parameters of DNJ, FGM, and DAB in rats after oral administration of SZ–A at 40, 200, 1000 mg/kg and intravenous injection of SZ–A at 4 mg/kg (*n* = 5).

Analyte	Group	C_max_	T_max_	t_1/2_	AUC_0–t_	AUC_0–∞_	Vd	CLz	MRT_0–t_	MRT_0–∞_	F
		(ng/mL)	(h)	(h)	(h * ng/mL)	(h * ng/mL)	(mL/kg)	(mL/h/kg)	(h)	(h)	(%)
DNJ	SZ–A 40 mg/kg (p.o.)	6370.0 ± 1116.9	0.67 ± 0.29	1.30 ± 0.25	10,114.3 ± 1412.0	10,153.0 ± 1444.6	2830.1 ± 741.1	1500.8 ± 195.5	1.71 ± 0.67	1.78 ± 0.69	72.41
	SZ–A 200 mg/kg (p.o.)	10,482.2 ± 2351.8	0.67 ± 0.20	2.11 ± 0.47	26,963.6 ± 3750.6	27,032.7 ± 3735.8	8617.8 ± 2317.5	2823.7 ± 401.6	2.63 ± 0.48	2.71 ± 0.49	38.61
	SZ–A 1000 mg/kg (p.o.)	25,090.5 ± 5126.1	0.43 ± 0.09	3.52 ± 0.85	116,270.3 ± 10547.8	116,695.9 ± 10709.1	16,468.3 ± 4217.0	3242.7 ± 298.1	5.12 ± 1.48	5.25 ± 1.53	33.29
	SZ–A 4 mg/kg (i.v.)	6083. 1 ± 1549.9	0.033	0.29 ± 0.08	1396.9 ± 174.5	1399.2 ± 174.6	453.4 ± 130.4	1086.5 ± 127.7	0.80 ± 0.50	0.82 ± 0.51	
	DNJ 15 mg/kg (p.o.)	4863.3 ± 1116.4	0.67 ± 0.29	0.88 ± 0.14 *	6731.5 ± 1146.2 **	6758.6 ± 1124. 1 **	2826.9 ± 347. 8	2261.6 ± 382.4 *	1.77 ± 0.25	1.85 ± 0.21	59.36
	DNJ 1.5 mg/kg (i.v.)	4522.4 ± 576.1 ^#^	0.03	0.25 ± 0.05	1133.9 ± 108.1 ^##^	1137.2 ± 108.4 ^##^	486.1 ± 85.40	1332.6 ± 128.4 ^#^	0.48 ± 0.08	0.50 ± 0.08	
FGM	SZ–A 40 mg/kg (p.o.)	1489.7 ± 756.0	0.67 ± 0.29	0.93 ± 0.29	2156.9 ± 778.3	2170.2 ± 770.4	1894.0 ± 1357.8	1317.9 ± 584.9	1.41 ± 0.27	1.48 ± 0.34	77.50
	SZ–A 200 mg/kg (p.o.)	2246.6 ± 1064.4	0.57 ± 0.09	1.44 ± 0.16	5355.7 ± 1489.8	5404.0 ± 1471.7	5332.0 ± 1855.9	2557.5 ± 786.6	2.66 ± 0.52	2.78 ± 0.54	38.49
	SZ–A 1000 mg/kg (p.o.)	4388.6 ± 2361.4	1.30 ± 0.67	2.73 ± 1.16	20,801.4 ± 7026.0	21,753.6 ± 6005.6	14,243.4 ± 9987.6	3385.8 ± 1262.0	5.44 ± 1.77	6.15 ± 2.11	29.90
	SZ–A 4 mg/kg (i.v.)	1240.4 ± 304.0	0.033	0.23 ± 0.03	278.3 ± 36.81	284.2 ± 36.69	308.1 ± 60.98	922.8 ± 109.5	0.26 ± 0.06	0.30 ± 0.06	
DAB	SZ–A 40 mg/kg (p.o.)	708.0 ± 171.3	0.67 ± 0.29	1.22 ± 0.16	968.9 ± 147.2	978.2 ± 140.5	3548.6 ± 990.1	1991.3 ± 298.6	1.32 ± 0.18	1.42 ± 0.25	78.23
	SZ–A 200 mg/kg (p.o.)	1997.3 ± 655.7	0.57 ± 0.09	1.38 ± 0.23	3506.2 ± 716.0	3555.0 ± 729.0	5472.4 ± 571.7	2815.7 ± 563.4	1.95 ± 0.35	2.09 ± 0.29	56.62
	SZ–A 1000 mg/kg (p.o.)	3771. 4 ± 1457.8	0.47 ± 0.14	2.38 ± 0.97	12,412.0 ± 2823.4	12,946.9 ± 2691.1	13,892.2 ± 7160.0	3926.0 ± 882.8	4.09 ± 1.19	4.66 ± 1.52	40.08
	SZ–A 4 mg/kg (i.v.)	624.9 ± 182.7	0.033	0.18 ± 0.03	123.9 ± 20.82	128.6 ± 20.07	400.7 ± 40.84	1535.6 ± 239.2	0.18 ± 0.04	0.21 ± 0.05	

Compared DNJ 15 mg/kg (p.o.) group with SZ–A 40 mg/kg (p.o.) group, * *p* < 0.05; ** *p* < 0.01; Compared DNJ 1.5 mg/kg (i.v.) group with SZ–A 4 mg/kg (i.v.) group, ^#^ *p* < 0.05, ^##^ *p* < 0.01.
